# (*E*)-4-{[(3-Propyl-5-sulfanyl­idene-4,5-dihydro-1*H*-1,2,4-triazol-4-yl)imino]­meth­yl}-3-(*p*-tol­yl)-1,2,3-oxadiazol-3-ium-5-olate

**DOI:** 10.1107/S1600536811037287

**Published:** 2011-09-17

**Authors:** Hoong-Kun Fun, Ching Kheng Quah, Nithinchandra, Balakrishna Kalluraya

**Affiliations:** a X-ray Crystallography Unit, School of Physics, Universiti Sains Malaysia, 11800 USM, Penang, Malaysia; b Department of Studies in Chemistry, Mangalore University, Mangalagangotri, Mangalore 574 199, India

## Abstract

The title compound, C_15_H_16_N_6_O_2_S, exists in a *trans* configuration with respect to the acyclic N=C bond. The 1,2,3-oxadiazol-3-ium ring makes dihedral angles of 10.59 (8) and 73.94 (8)°, respectively, with the 1,2,4-triazole and benzene rings. The mol­ecular structure is stabilized by an intra­molecular C—H⋯S hydrogen bond, which generates an *S*(6) ring motif. In the crystal, mol­ecules are linked into inversion dimers by pairs of inter­molecular N—H⋯S hydrogen bonds, generating eight-membered *R*_2_^2^(8) ring motifs. The dimers are further connected by C—H⋯O hydrogen bonds, forming a sheet parallel to the *bc* plane. The ethyl group is disordered over two sets of sites with occupancies of 0.744 (7) and 0.256 (7).

## Related literature

For general background to and applications of sydnone derivatives, see: Baker *et al.* (1949 ▶); Hedge *et al.* (2008 ▶); Rai *et al.* (2008 ▶); Kalluraya *et al.* (2002 ▶). For standard bond-length data, see: Allen *et al.* (1987 ▶). For graph-set notation, see: Bernstein *et al.* (1995 ▶). For a related structure, see: Fun *et al.* (2011 ▶).
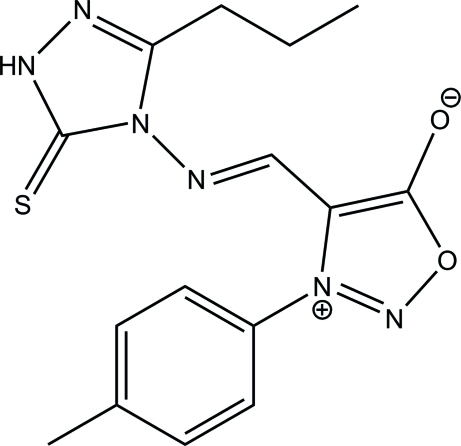


## Experimental

### 

#### Crystal data

C_15_H_16_N_6_O_2_S
*M* *_r_* = 344.40
Monoclinic, 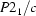

*a* = 13.4220 (11) Å
*b* = 6.2411 (5) Å
*c* = 21.1374 (16) Å
β = 104.575 (2)°
*V* = 1713.7 (2) Å^3^
*Z* = 4
Mo *K*α radiation
μ = 0.21 mm^−1^
*T* = 296 K
0.51 × 0.17 × 0.08 mm

#### Data collection

Bruker SMART APEXII DUO CCD area-detector diffractometer
Absorption correction: multi-scan (*SADABS*; Bruker, 2009 ▶) *T*_min_ = 0.865, *T*_max_ = 0.983
18912 measured reflections
5003 independent reflections
3637 reflections with *I* > 2σ(*I*)
*R*_int_ = 0.030

#### Refinement

*R*[*F* ^2^ > 2σ(*F* ^2^)] = 0.039
*wR*(*F* ^2^) = 0.123
*S* = 1.04
5003 reflections
243 parameters
H atoms treated by a mixture of independent and constrained refinement
Δρ_max_ = 0.22 e Å^−3^
Δρ_min_ = −0.21 e Å^−3^

### 

Data collection: *APEX2* (Bruker, 2009 ▶); cell refinement: *SAINT* (Bruker, 2009 ▶); data reduction: *SAINT*; program(s) used to solve structure: *SHELXTL* (Sheldrick, 2008 ▶); program(s) used to refine structure: *SHELXTL*; molecular graphics: *SHELXTL*; software used to prepare material for publication: *SHELXTL* and *PLATON* (Spek, 2009 ▶).

## Supplementary Material

Crystal structure: contains datablock(s) global, I. DOI: 10.1107/S1600536811037287/is2776sup1.cif

Structure factors: contains datablock(s) I. DOI: 10.1107/S1600536811037287/is2776Isup2.hkl

Supplementary material file. DOI: 10.1107/S1600536811037287/is2776Isup3.cml

Additional supplementary materials:  crystallographic information; 3D view; checkCIF report

## Figures and Tables

**Table 1:** Hydrogen-bond geometry (Å, °)

*D*—H⋯*A*	*D*—H	H⋯*A*	*D*⋯*A*	*D*—H⋯*A*
N5—H1*N*5⋯S1^i^	0.857 (18)	2.440 (18)	3.2933 (13)	174.3 (16)
C1—H1*A*⋯O2^ii^	0.93	2.48	3.346 (2)	154
C9—H9*A*⋯S1	0.93	2.42	3.1845 (13)	139

Symmetry codes: (i) 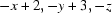; (ii) 
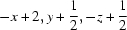
.

**Fig. 1.**
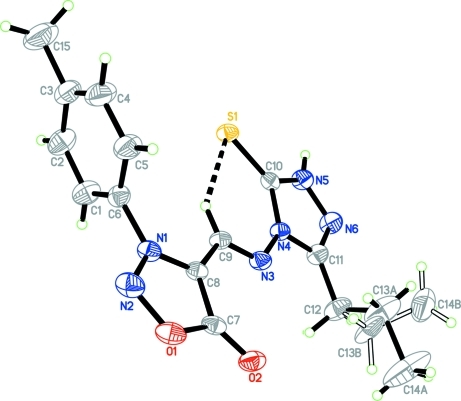
The molecular structure of the title compound showing 30% probability displacement ellipsoids for non-H atoms. The intramolecular hydrogen bond is shown as a dashed line. The minor component of disorder is shown as open bonds.

**Fig. 2.**
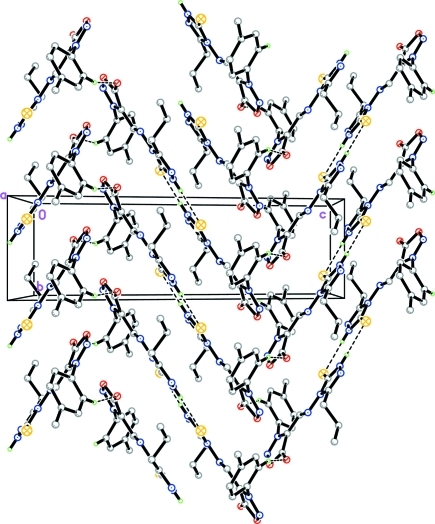
The crystal structure of the title compound, viewed along the a axis. H atoms not involved in hydrogen bonds (dashed lines) have been omitted for clarity. Only the major disorder component is shown.

## supplementary crystallographic information

### Comment

Sydnones constitute a well defined class of mesoionic compounds consisting of 1,2,3-oxadiazole ring system. The introduction of the concept of mesoionic structure for certain heterocyclic compounds in the year 1949

has proved to be a fruitful development in heterocyclic chemistry (Baker *et al.*, 1949). The study of sydnones still remains a field of interest because of their electronic structures and also because of the various types of biological activities displayed by some of them. Interest in sydnone derivatives has also been encouraged by the discovery that they exhibit various pharmacological activities (Hedge *et al.*, 2008; Rai *et al.*, 2008). The 4-formyl sydnone will be used for the preparation of a new series of Schiff bases by condensation with appropriate 4-amino-4H-1,2,4-triazole-3-thiol. These Schiff bases containing sydnone is utilized for the synthesis of appropriate Mannich bases (Kalluraya *et al.*, 2002).

The molecular structure is shown in Fig. 1. Bond lengths (Allen *et al.*, 1987) and angles are within normal ranges and are comparable to a related structure (Fun *et al.*, 2011). The title compound exists in *trans*  configuration with respect to the acyclic N3═C9 bond [bond lengths = 1.2669 (17) Å]. The 1,2,4-triazole (N4–N6/C10/C11, maximum deviation of 0.004 (1) Å at atom N4) and the phenyl (C1–C6) rings form dihedral angles of 73.94 (8) and 10.59 (8)°, respectively, with the 1,2,3-oxadiazol-3-ium ring (O1/N1/N2/C7/C8, maximum deviation of 0.004 (1) Å at atoms C7 and C8). The molecular structure is stabilized by an intramolecular C9–H9A···S1 hydrogen bond, which generates an *S*(6) ring motif (Fig. 1, Bernstein *et al.*, 1995). The ethyl group is disordered over two sets of sites in a 0.744 (7): 0.256 (7) ratio.

In the crystal (Fig. 2), the intermolecular N5—H1N5···S1 hydrogen bonds (Table 1) form the inversion dimers and produce eight-membered ring motifs R^2^_2_(8) (Bernstein *et al.*, 1995). Another intermolecular C1—H1A···O2 hydrogen bond connects these dimers to another molecule forming two-dimensional sheets parallel to the *bc* plane.

### Experimental

4-Formyl-3-*p*-tolylsydnone (0.01 mol) and 4-amino-5-propyl-4*H*-1,2,4-triazole-3-thiol (0.01 mol) in ethanol and a catalytic amount of conc. sulphuric acid was stirred at room temperature for 2-3 h. The solid product that separated out was filtered and dried. It was then recrystallized from ethanol. Crystals suitable for X-ray analysis were obtained from a 1:2 mixture solution of DMF and ethanol by slow evaporation.

### Refinement

Atom H1N5 was located in a difference Fourier map and refined freely [N5—H1N5 = 0.855 (18) Å]. The remaining H atoms were positioned geometrically and refined using a riding model, with C—H = 0.93–0.97 Å and *U*_iso_(H) = 1.2 or 1.5*U*_eq_(C). A rotating-group model was applied for the methyl groups. The ethyl group is disordered over two sets of sites in a 0.744 (7): 0.256 (7) ratio.

### Crystal data

**Table:**

C_15_H_16_N_6_O_2_S	*F*(000) = 720
*M**_r_* = 344.40	*D*_x_ = 1.335 Mg m^−^^3^
Monoclinic, *P*2_1_/*c*	Mo *K*α radiation, λ = 0.71073 Å
Hall symbol: -P 2ybc	Cell parameters from 5356 reflections
*a* = 13.4220 (11) Å	θ = 2.2–27.6°
*b* = 6.2411 (5) Å	µ = 0.21 mm^−^^1^
*c* = 21.1374 (16) Å	*T* = 296 K
β = 104.575 (2)°	Needle, colourless
*V* = 1713.7 (2) Å^3^	0.51 × 0.17 × 0.08 mm
*Z* = 4	

### Data collection

**Table:**

Bruker SMART APEXII DUO CCD area-detector diffractometer	5003 independent reflections
Radiation source: fine-focus sealed tube	3637 reflections with *I* > 2σ(*I*)
graphite	*R*_int_ = 0.030
φ and ω scans	θ_max_ = 30.1°, θ_min_ = 2.0°
Absorption correction: multi-scan (*SADABS*; Bruker, 2009)	*h* = −18→18
*T*_min_ = 0.865, *T*_max_ = 0.983	*k* = −8→8
18912 measured reflections	*l* = −29→29

### Refinement

**Table:**

Refinement on *F*^2^	Primary atom site location: structure-invariant direct methods
Least-squares matrix: full	Secondary atom site location: difference Fourier map
*R*[*F*^2^ > 2σ(*F*^2^)] = 0.039	Hydrogen site location: inferred from neighbouring sites
*wR*(*F*^2^) = 0.123	H atoms treated by a mixture of independent and constrained refinement
*S* = 1.04	*w* = 1/[σ^2^(*F*_o_^2^) + (0.0622*P*)^2^ + 0.2043*P*] where *P* = (*F*_o_^2^ + 2*F*_c_^2^)/3
5003 reflections	(Δ/σ)_max_ = 0.001
243 parameters	Δρ_max_ = 0.22 e Å^−^^3^
0 restraints	Δρ_min_ = −0.21 e Å^−^^3^

### Special details

**Table:**

Geometry. All esds (except the esd in the dihedral angle between two l.s. planes) are estimated using the full covariance matrix. The cell esds are taken into account individually in the estimation of esds in distances, angles and torsion angles; correlations between esds in cell parameters are only used when they are defined by crystal symmetry. An approximate (isotropic) treatment of cell esds is used for estimating esds involving l.s. planes.
Refinement. Refinement of F^2^ against ALL reflections. The weighted R-factor wR and goodness of fit S are based on F^2^, conventional R-factors R are based on F, with F set to zero for negative F^2^. The threshold expression of F^2^ > 2sigma(F^2^) is used only for calculating R-factors(gt) etc. and is not relevant to the choice of reflections for refinement. R-factors based on F^2^ are statistically about twice as large as those based on F, and R- factors based on ALL data will be even larger.

### Fractional atomic coordinates and isotropic or equivalent isotropic displacement parameters (Å^2^)

**Table:**

	*x*	*y*	*z*	*U*_iso_*/*U*_eq_	Occ. (<1)
S1	1.08849 (3)	1.22661 (6)	0.061779 (16)	0.04353 (11)	
O1	1.07417 (9)	0.32936 (18)	0.22957 (5)	0.0546 (3)	
O2	0.90860 (8)	0.41866 (18)	0.18192 (5)	0.0532 (3)	
N1	1.15527 (9)	0.59146 (19)	0.20345 (5)	0.0412 (3)	
N2	1.17089 (11)	0.4104 (2)	0.23528 (7)	0.0560 (3)	
N3	0.93160 (9)	0.8734 (2)	0.11642 (5)	0.0419 (3)	
N4	0.90503 (8)	1.05639 (18)	0.07899 (5)	0.0372 (2)	
N5	0.88806 (10)	1.3345 (2)	0.01976 (6)	0.0487 (3)	
N6	0.78985 (10)	1.2811 (2)	0.02200 (7)	0.0552 (3)	
C1	1.26773 (13)	0.9030 (3)	0.23096 (9)	0.0590 (4)	
H1A	1.2297	0.9496	0.2595	0.071*	
C2	1.34955 (13)	1.0220 (3)	0.22159 (10)	0.0661 (5)	
H2A	1.3667	1.1500	0.2443	0.079*	
C3	1.40617 (13)	0.9549 (4)	0.17941 (9)	0.0676 (5)	
C4	1.37997 (16)	0.7634 (4)	0.14644 (10)	0.0825 (7)	
H4A	1.4184	0.7155	0.1182	0.099*	
C5	1.29812 (14)	0.6417 (4)	0.15446 (9)	0.0684 (5)	
H5A	1.2804	0.5142	0.1316	0.082*	
C6	1.24376 (11)	0.7144 (2)	0.19713 (7)	0.0443 (3)	
C7	0.99772 (11)	0.4668 (2)	0.19272 (6)	0.0411 (3)	
C8	1.05554 (10)	0.6411 (2)	0.17668 (6)	0.0361 (3)	
C9	1.02620 (10)	0.8309 (2)	0.13889 (6)	0.0386 (3)	
H9A	1.0762	0.9226	0.1308	0.046*	
C10	0.96113 (10)	1.2042 (2)	0.05372 (6)	0.0373 (3)	
C11	0.80236 (11)	1.1116 (3)	0.05860 (7)	0.0474 (3)	
C12	0.71870 (12)	0.9897 (3)	0.07693 (11)	0.0679 (5)	
H12A	0.6594	1.0797	0.0726	0.081*	0.744 (7)
H12B	0.7412	0.9443	0.1217	0.081*	0.744 (7)
H12C	0.6567	1.0756	0.0638	0.081*	0.256 (7)
H12D	0.7358	0.9814	0.1243	0.081*	0.256 (7)
C13A	0.6932 (7)	0.7918 (14)	0.0331 (4)	0.102 (3)	0.744 (7)
H13A	0.7545	0.7043	0.0384	0.123*	0.744 (7)
H13B	0.6718	0.8368	−0.0122	0.123*	0.744 (7)
C14A	0.6070 (3)	0.6572 (8)	0.0496 (3)	0.164 (3)	0.744 (7)
H14A	0.6042	0.5194	0.0290	0.245*	0.744 (7)
H14B	0.5423	0.7291	0.0340	0.245*	0.744 (7)
H14C	0.6209	0.6390	0.0961	0.245*	0.744 (7)
C13B	0.6925 (14)	0.770 (4)	0.0589 (11)	0.082 (6)	0.256 (7)
H13C	0.6474	0.7109	0.0839	0.098*	0.256 (7)
H13D	0.7538	0.6814	0.0658	0.098*	0.256 (7)
C14B	0.6399 (10)	0.782 (2)	−0.0107 (6)	0.111 (5)	0.256 (7)
H14D	0.6068	0.6475	−0.0247	0.166*	0.256 (7)
H14E	0.6892	0.8125	−0.0354	0.166*	0.256 (7)
H14F	0.5892	0.8937	−0.0175	0.166*	0.256 (7)
C15	1.49528 (18)	1.0890 (5)	0.16902 (14)	0.1059 (9)	
H15A	1.4908	1.2311	0.1855	0.159*	
H15B	1.4921	1.0958	0.1232	0.159*	
H15C	1.5592	1.0247	0.1919	0.159*	
H1N5	0.8984 (13)	1.445 (3)	−0.0018 (8)	0.053 (5)*	

### Atomic displacement parameters (Å^2^)

**Table:**

	*U*^11^	*U*^22^	*U*^33^	*U*^12^	*U*^13^	*U*^23^
S1	0.04646 (19)	0.0376 (2)	0.04930 (19)	−0.00191 (14)	0.01724 (14)	0.00811 (15)
O1	0.0733 (7)	0.0373 (6)	0.0597 (6)	0.0047 (5)	0.0290 (5)	0.0156 (5)
O2	0.0634 (7)	0.0455 (6)	0.0582 (6)	−0.0161 (5)	0.0295 (5)	−0.0057 (5)
N1	0.0470 (6)	0.0360 (6)	0.0430 (5)	0.0075 (5)	0.0159 (5)	0.0074 (5)
N2	0.0652 (8)	0.0450 (8)	0.0605 (7)	0.0138 (6)	0.0207 (6)	0.0192 (6)
N3	0.0436 (6)	0.0370 (6)	0.0467 (6)	0.0015 (5)	0.0143 (5)	0.0112 (5)
N4	0.0403 (5)	0.0317 (6)	0.0409 (5)	0.0015 (4)	0.0127 (4)	0.0046 (4)
N5	0.0514 (7)	0.0394 (7)	0.0548 (7)	0.0039 (6)	0.0128 (5)	0.0145 (6)
N6	0.0474 (7)	0.0497 (8)	0.0666 (8)	0.0070 (6)	0.0108 (6)	0.0151 (7)
C1	0.0542 (8)	0.0500 (10)	0.0791 (10)	0.0052 (7)	0.0286 (8)	−0.0071 (8)
C2	0.0557 (9)	0.0518 (10)	0.0933 (13)	−0.0060 (8)	0.0234 (9)	−0.0122 (10)
C3	0.0485 (9)	0.0806 (14)	0.0764 (11)	−0.0124 (9)	0.0203 (8)	−0.0046 (10)
C4	0.0672 (11)	0.1099 (19)	0.0830 (12)	−0.0267 (12)	0.0424 (10)	−0.0313 (13)
C5	0.0622 (10)	0.0821 (14)	0.0679 (10)	−0.0176 (10)	0.0293 (8)	−0.0269 (10)
C6	0.0396 (6)	0.0440 (8)	0.0494 (7)	0.0044 (6)	0.0115 (5)	0.0046 (6)
C7	0.0599 (8)	0.0309 (7)	0.0384 (6)	−0.0021 (6)	0.0234 (5)	−0.0015 (5)
C8	0.0436 (6)	0.0301 (6)	0.0379 (6)	0.0011 (5)	0.0161 (5)	0.0017 (5)
C9	0.0425 (6)	0.0309 (6)	0.0454 (6)	−0.0014 (5)	0.0167 (5)	0.0051 (5)
C10	0.0480 (7)	0.0305 (6)	0.0346 (5)	0.0000 (5)	0.0126 (5)	0.0005 (5)
C11	0.0424 (7)	0.0443 (8)	0.0555 (8)	0.0043 (6)	0.0119 (6)	0.0050 (7)
C12	0.0438 (8)	0.0637 (12)	0.0984 (13)	0.0038 (8)	0.0222 (8)	0.0150 (11)
C13A	0.079 (3)	0.067 (4)	0.172 (8)	−0.012 (3)	0.054 (5)	−0.010 (4)
C14A	0.109 (3)	0.099 (3)	0.316 (8)	−0.047 (3)	0.115 (4)	−0.049 (4)
C13B	0.045 (5)	0.052 (6)	0.137 (15)	−0.018 (4)	0.004 (7)	0.034 (9)
C14B	0.080 (7)	0.132 (11)	0.102 (8)	−0.029 (7)	−0.013 (6)	0.001 (7)
C15	0.0746 (14)	0.125 (2)	0.130 (2)	−0.0437 (15)	0.0464 (14)	−0.0228 (18)

### Geometric parameters (Å, °)

**Table:**

S1—C10	1.6805 (14)	C7—C8	1.4260 (18)
O1—N2	1.3698 (18)	C8—C9	1.4273 (18)
O1—C7	1.4118 (18)	C9—H9A	0.9300
O2—C7	1.1982 (17)	C11—C12	1.486 (2)
N1—N2	1.3051 (17)	C12—C13B	1.45 (2)
N1—C8	1.3515 (17)	C12—C13A	1.530 (10)
N1—C6	1.4482 (18)	C12—H12A	0.9600
N3—C9	1.2669 (17)	C12—H12B	0.9600
N3—N4	1.3838 (16)	C12—H12C	0.9700
N4—C11	1.3800 (17)	C12—H12D	0.9700
N4—C10	1.3806 (16)	C13A—C14A	1.539 (8)
N5—C10	1.3355 (19)	C13A—H13A	0.9700
N5—N6	1.3720 (18)	C13A—H13B	0.9700
N5—H1N5	0.855 (18)	C14A—H14A	0.9600
N6—C11	1.296 (2)	C14A—H14B	0.9600
C1—C6	1.373 (2)	C14A—H14C	0.9600
C1—C2	1.381 (2)	C13B—C14B	1.46 (2)
C1—H1A	0.9300	C13B—H13C	0.9700
C2—C3	1.374 (3)	C13B—H13D	0.9700
C2—H2A	0.9300	C14B—H14D	0.9600
C3—C4	1.384 (3)	C14B—H14E	0.9600
C3—C15	1.520 (3)	C14B—H14F	0.9600
C4—C5	1.381 (3)	C15—H15A	0.9600
C4—H4A	0.9300	C15—H15B	0.9600
C5—C6	1.372 (2)	C15—H15C	0.9600
C5—H5A	0.9300		
			
N2—O1—C7	111.44 (10)	N6—C11—C12	125.49 (14)
N2—N1—C8	115.36 (12)	N4—C11—C12	123.46 (14)
N2—N1—C6	118.51 (12)	C13B—C12—C11	124.6 (8)
C8—N1—C6	126.06 (12)	C11—C12—C13A	108.9 (3)
N1—N2—O1	104.38 (11)	C13B—C12—H12A	113.0
C9—N3—N4	118.50 (11)	C11—C12—H12A	109.8
C11—N4—C10	108.14 (11)	C13A—C12—H12A	111.1
C11—N4—N3	118.55 (11)	C13B—C12—H12B	88.6
C10—N4—N3	133.28 (11)	C11—C12—H12B	109.7
C10—N5—N6	114.54 (13)	C13A—C12—H12B	109.0
C10—N5—H1N5	125.4 (12)	H12A—C12—H12B	108.4
N6—N5—H1N5	120.0 (12)	C13B—C12—H12C	108.4
C11—N6—N5	103.75 (12)	C11—C12—H12C	107.0
C6—C1—C2	118.49 (15)	C13A—C12—H12C	103.1
C6—C1—H1A	120.8	H12B—C12—H12C	118.7
C2—C1—H1A	120.8	C13B—C12—H12D	101.6
C3—C2—C1	121.25 (18)	C11—C12—H12D	107.3
C3—C2—H2A	119.4	C13A—C12—H12D	122.7
C1—C2—H2A	119.4	H12A—C12—H12D	96.0
C2—C3—C4	118.55 (17)	H12C—C12—H12D	106.8
C2—C3—C15	120.6 (2)	C12—C13A—C14A	111.7 (6)
C4—C3—C15	120.82 (18)	C12—C13A—H13A	109.3
C5—C4—C3	121.52 (17)	C14A—C13A—H13A	109.3
C5—C4—H4A	119.2	C12—C13A—H13B	109.3
C3—C4—H4A	119.2	C14A—C13A—H13B	109.3
C6—C5—C4	118.02 (18)	H13A—C13A—H13B	107.9
C6—C5—H5A	121.0	C12—C13B—C14B	103.8 (12)
C4—C5—H5A	121.0	C12—C13B—H13C	111.0
C5—C6—C1	122.16 (15)	C14B—C13B—H13C	111.0
C5—C6—N1	118.00 (14)	C12—C13B—H13D	111.0
C1—C6—N1	119.76 (13)	C14B—C13B—H13D	111.0
O2—C7—O1	120.32 (12)	H13C—C13B—H13D	109.0
O2—C7—C8	136.30 (14)	C13B—C14B—H14D	109.5
O1—C7—C8	103.37 (12)	C13B—C14B—H14E	109.5
N1—C8—C7	105.45 (12)	H14D—C14B—H14E	109.5
N1—C8—C9	121.96 (12)	C13B—C14B—H14F	109.5
C7—C8—C9	132.54 (12)	H14D—C14B—H14F	109.5
N3—C9—C8	119.54 (12)	H14E—C14B—H14F	109.5
N3—C9—H9A	120.2	C3—C15—H15A	109.5
C8—C9—H9A	120.2	C3—C15—H15B	109.5
N5—C10—N4	102.51 (12)	H15A—C15—H15B	109.5
N5—C10—S1	126.47 (11)	C3—C15—H15C	109.5
N4—C10—S1	131.01 (10)	H15A—C15—H15C	109.5
N6—C11—N4	111.05 (13)	H15B—C15—H15C	109.5
			
C8—N1—N2—O1	0.29 (16)	O1—C7—C8—N1	0.69 (13)
C6—N1—N2—O1	−176.84 (11)	O2—C7—C8—C9	−1.1 (3)
C7—O1—N2—N1	0.21 (15)	O1—C7—C8—C9	177.93 (13)
C9—N3—N4—C11	−175.21 (13)	N4—N3—C9—C8	−178.92 (11)
C9—N3—N4—C10	7.3 (2)	N1—C8—C9—N3	−177.96 (12)
C10—N5—N6—C11	−0.05 (18)	C7—C8—C9—N3	5.2 (2)
C6—C1—C2—C3	0.1 (3)	N6—N5—C10—N4	0.44 (16)
C1—C2—C3—C4	−0.4 (3)	N6—N5—C10—S1	−179.18 (11)
C1—C2—C3—C15	179.3 (2)	C11—N4—C10—N5	−0.64 (14)
C2—C3—C4—C5	0.9 (3)	N3—N4—C10—N5	177.05 (13)
C15—C3—C4—C5	−178.8 (2)	C11—N4—C10—S1	178.95 (11)
C3—C4—C5—C6	−1.0 (3)	N3—N4—C10—S1	−3.4 (2)
C4—C5—C6—C1	0.7 (3)	N5—N6—C11—N4	−0.38 (17)
C4—C5—C6—N1	177.43 (18)	N5—N6—C11—C12	179.64 (16)
C2—C1—C6—C5	−0.2 (3)	C10—N4—C11—N6	0.68 (17)
C2—C1—C6—N1	−176.94 (15)	N3—N4—C11—N6	−177.41 (12)
N2—N1—C6—C5	73.94 (19)	C10—N4—C11—C12	−179.35 (15)
C8—N1—C6—C5	−102.85 (18)	N3—N4—C11—C12	2.6 (2)
N2—N1—C6—C1	−109.20 (17)	N6—C11—C12—C13B	116.8 (9)
C8—N1—C6—C1	74.02 (19)	N4—C11—C12—C13B	−63.2 (9)
N2—O1—C7—O2	178.68 (12)	N6—C11—C12—C13A	100.0 (4)
N2—O1—C7—C8	−0.57 (14)	N4—C11—C12—C13A	−80.0 (4)
N2—N1—C8—C7	−0.65 (15)	C13B—C12—C13A—C14A	40 (2)
C6—N1—C8—C7	176.23 (12)	C11—C12—C13A—C14A	179.8 (5)
N2—N1—C8—C9	−178.25 (12)	C11—C12—C13B—C14B	−74.1 (13)
C6—N1—C8—C9	−1.4 (2)	C13A—C12—C13B—C14B	−26 (2)
O2—C7—C8—N1	−178.37 (15)		

### Hydrogen-bond geometry (Å, °)

**Table:**

*D*—H···*A*	*D*—H	H···*A*	*D*···*A*	*D*—H···*A*
N5—H1N5···S1^i^	0.857 (18)	2.440 (18)	3.2933 (13)	174.3 (16)
C1—H1A···O2^ii^	0.93	2.48	3.346 (2)	154
C9—H9A···S1	0.93	2.42	3.1845 (13)	139

Symmetry codes: (i) −*x*+2, −*y*+3, −*z*; (ii) −*x*+2, *y*+1/2, −*z*+1/2.
